# Remaining useful lifetime estimation for discrete power electronic devices using physics-informed neural network

**DOI:** 10.1038/s41598-023-37154-5

**Published:** 2023-06-22

**Authors:** Zhonghai Lu, Chao Guo, Mingrui Liu, Rui Shi

**Affiliations:** Department of Electrical Engineering, KTH Royal Institute of Technology, 16440 Stockholm, Sweden

**Keywords:** Electrical and electronic engineering, Computer science

## Abstract

Estimation of Remaining Useful Lifetime (RUL) of discrete power electronics is important to enable predictive maintenance and ensure system safety. Conventional data-driven approaches using neural networks have been applied to address this challenge. However, due to ignoring the physical properties of the target RUL function, neural networks can result in unreasonable RUL estimates such as going upwards and wrong endings. In the paper, we apply the fundamental principle of Physics-Informed Neural Network (PINN) to enhance Recurrent Neural Network (RNN) based RUL estimation methods. Through formulating proper constraints into the loss function of neural networks, we demonstrate in our experiments with the NASA IGBT dataset that PINN can make the neural networks trained more realistically and thus achieve performance improvements in estimation error and coefficient of determination. Compared to the baseline vanilla RNN, our physics-informed RNN can improve Mean Squared Error (MSE) of out-of-sample estimation on average by 24.7% in training and by 51.3% in testing; Compared to the baseline Long Short Term Memory (LSTM, a variant of RNN), our physics-informed LSTM can improve MSE of out-of-sample estimation on average by 15.3% in training and 13.9% in testing.

## Introduction

Discrete power electronic devices like Insulated-Gate Bipolar Transistors (IGBTs) are widely used in power management circuits (power switching, rectifying, etc.) of safety-critical application domains such as automotive, locomotive, aerospace, and power grids, etc. To enable prognostic health management, one key element is to be able to estimate their Remaining Useful Lifetime (RUL) in actual operations.

Due to the rising interest in deep learning^1^, Neural Network (NN) has been a popular data-driven approach to studying the IGBT RUL prediction problem. Various NNs such as Multi-Layer Perceptron (MLP)^2^, Long Short Term Memory (LSTM)^3^, Attention Neural Network^4^, etc., have been tried to address this problem. Despite showing promises, NN-based methods can sometimes produce non-realistic estimates, for example, values going up or even becoming negative in extreme cases. This is inconsistent with the target RUL function, which is monotonically decreasing till zero at the end. This phenomenon occurs in RUL estimation of IGBT^5^ and other components such as turbofan engine^6,7^ and bearing^8,9^, etc.

Physics-Informed Neural Network (PINN) has been proposed to enhance pure data-driven neural networks with physical rules^10,11^. The fundamental principle of PINN is to formulate physical rules based constraints into the loss function of NNs, such that the NNs can be trained to respect physical conditions rather than arbitrarily optimizing parameters in the training process. This is appealing because the trained NN models can generate more realistic results. PINN was initially proposed to solve mathematical problems^10,11^, such as ordinary and partial differential equations. Very recently it was also applied in the domain of power electronics for parameter estimation^12–15^. However, it has not been employed to address the RUL estimation problem of power electronic devices.

In the paper, we address the RUL estimation problem for IGBTs based on PINN. Our baseline is the RUL estimation method using RNN, being vanilla RNN (Recurrent Neural Network) or LSTM (a more powerful variant of RNN). We identify physical rules and formulate them as regularization terms into the loss function of the RNN to realize PINN-based RUL estimation. Our approach can overcome the potential mis-estimation behavior of NN-based estimation methods. The major contributions of the paper can be summarized as follows.

- For the first time, we apply the principle of PINN to estimate the RUL of discrete power electronic devices. In particular, we identify and formulate physical rules as mathematical conditions, and then embed them as regularization terms into the loss function of neural networks for RUL estimation.
- We demonstrate that our PINN-based RUL estimation method can improve the regression performance in both Mean Squared Error (MSE) and coefficient of determination, $R^2$, when compared to the baseline RNN, which can be either a vanilla RNN or an LSTM.

## Related work

Various methods have been developed for IGBT RUL/lifetime modeling and estimation. These methods can be broadly classified into *model based* approach^16–19^, *data-driven* approach^3,20,21^, and *hybrid* approach^22^.

The model-based approach uses an analytical formula to express the relationship between the device’s lifetime and its dependent factors. Analytical lifetime models^23^ are related to physics-of-failure models since their modeling processing is based on known failure modes under certain conditions^24,25^. These models estimate the number of cycles to failure ($N_f$) of the IGBT devices. There are several famous analytical lifetime models formulated on the assumption that plastic strain caused by the large thermo-mechanical mismatch between the adjacent packaging layers is the main reason for IGBT failure^24^: The Coffin–Manson model that only considers temperature swing ($\Delta T$) was the original model spawning all the following variant models^26^; The Coffin–Manson–Arrhenius model takes temperature amplitude of the junction temperature ($\Delta T_j$) and medium temperature ($T_m$) into consideration^16^, whereas a similar model is the LESIT equation^17^, which performs well on modeling the lifetime of discrete TO-2xx based power devices^27,28^; The Norris–Landzberg equation considers also the frequency of temperature cycles (*f*) in the model equation^18^; The Bayerer’s model contains a lot of parameters including maximum junction temperature ($T_{j,max}$), heating time ($t_{on}$), applied DC current (*I*), diameter of the bond wire (*D*), and blocking voltage (*V*)^19^. Cumulative damage models with rainflow counting algorithm are commonly accompanied by the analytical models to conduct the estimation^29^. Apart from the mainstream analytical models, physical lifetime models based on energy assumptions also exist^30^.

Unlike the model-based approach, the data-driven approach does not require the failure mode knowledge of the IGBT devices. It uses existing experimental data to train a regression machine-learning model for lifetime estimation using precursor signals as the feature vector^24^. Common IGBT failure precursor signals are collector current ($I_c$), collector-emitter ON-state voltage ($V_{ce, on}$), gate-emitter voltage ($V_{ge}$), gate-emitter threshold voltage ($V_{ge,th}$), junction temperature ($T_j$), switch turn on ($T_{on}$) and turn off ($T_{off}$) time, etc^20,24^. Various machine learning algorithms have been applied in various contexts. In statistical learning, Kalman filter algorithm was used to estimate the junction temperature in IGBT device^31^. Particle filter was adopted widely in estimating IGBT RUL^20,32^. A modified maximum likelihood estimator algorithm was developed and applied to IGBT RUL estimation problem^21^.

Besides statistical learning methods, NNs have also been applied to IGBT RUL prediction. It was shown in^33^ that NNs slightly outperformed a competitive counterpart, Adaptive Neuro Fuzzy Inference System (ANFIS). Efforts were also made on preprocessing the precursor signal data. Principal component analysis (PCA) was applied to the time domain features of the precursor signal before it was fed into a feed-forward NN^2^. More complex deep learning algorithms were investigated recently. LSTM was introduced to predict the RUL of IGBTs^3^. Compared with two model-based methods, the LSTM method achieves a higher accuracy, while a larger dataset is required to train the model. Attention mechanism was first applied to the IGBT RUL prognostics^4^.

To deal with package failure in solder joints, correlation-driven neural network (CDNN)^34^ and deep neural network^35^ were proposed to predict useful lifetime of solder joints in electronic devices. In, Samavatian et al.^36^ enhanced the CDNN method by establishing a novel iterative machine learning-aid framework to improve the useful lifetime prediction results.

A hybrid approach intends to fuse physical information into the model-building process of the data-driven approach. It is considered a promising approach as it can combine the advantages of both model-based and data-driven approaches. A particle filter based method incorporating the crack propagation physics law was developed to predict the RUL of IGBT modules^22^. A hybrid framework adopting the PINN idea was proposed^37^. The framework achieves better results on the turbofan engine RUL prediction than the pure data-driven approach with less training data required.

Very recently, the principle of PINN has also been applied to the domain of power electronics. In^12^, PINN was used to estimate the parameters of the DC-DC buck converter. In^13^, PINN was adopted to evaluate the impedance of voltage source converter by means of its physics knowledge. In, Wu et al.^14^ proposed AutoPINN, a combination of AutoML and PINN, that can automatically design PINN models. It shows that the PINN model designed by AutoPINN reaches better performance in the parameter estimation of power electronic converters. Chen et al.^15^ proposed PI-LSTM, which is a combination of PINN and LSTM, to estimate the parameters of DC-DC buck converter.

Our work may belong to the hybrid approach for IGBT RUL estimation. It advances the current data-driven NN-based approach by incorporating physical rules into the network training. The physical rules are directly derived from the target RUL function. It can overcome possible drawbacks (estimated values going up or becoming negative in extreme cases) of the pure data-driven NN-based approach and further enhance its performance.

## The RUL estimation problem and associated physical rules

To overcome the practical difficulty in collecting data from realtime operations, RUL estimation has largely resorted to utilizing experimental data from aggregated aging tests. In our study, we use the IGBT aggregated aging test dataset from NASA Prognostics Center of Excellence (PCoE)^38^. With this dataset, we can consider the collector-emitter voltage $V_{ce}$ as the precursor signal to capture the transistor latch-up failure^39^, and use it to estimate the device RUL, as prior studies^2,5,33^ did. Informally, the RUL estimation problem aims to find a mapping relationship from a collector-emitter voltage $V_{ce}$ series to its corresponding RUL series. We can formulate the problem as follows.

Given is a collector-emitter voltage series $\{V_{ce}(t)\}$, where $V_{ce}(t)\in {\mathcal {R}}$, $t\in [0, N_f]$; *t* is in (test) cycle, and $N_f$ is the lifetime of the device. When $t\ge N_f$, the device fails. The goal of RUL estimation is to find a mapping function ${\mathcal {F}}$ such that $V_{ce}(t) \rightarrow {\hat{RUL}}(t)$, i.e., ${\hat{RUL}}(t)={\mathcal {F}}(V_{ce}(t))$, where ${\hat{RUL}}(t)\in {\mathbb {N}}$, $t\in [0, N_f]$, is the estimated RUL series.

The RUL estimation problem can be treated as a supervised learning problem, meaning that there is a target RUL series *RUL*(*t*). As of now, the commonly used target RUL function *RUL*(*t*) is a simple linear function, starting from a normalized value of 1 down to 0. It can be mathematically expressed as follows.

1 $$\begin{aligned} RUL(t)= 1 - t/N_f. \end{aligned}$$

In this equation, the exact failure time, $N_f$, is unknown and thus it is impossible to determine the end of lifetime $(RUL(t=N_f)=0)$. However, in our RUL estimation, we consider a relative (not absolute) lifetime. In Eq. 1, the term $t/N_f$ is a fraction of total lifetime $N_f$. Then we can derive the following two physical rules or properties directly from Eq. (1):

1. Monotonic decreasing condition: There is a monotonously decreasing linear relationship between *t* and *RUL*(*t*), even though the degradation rate $1/N_f$ may vary from device to device.
2. Boundary condition: Apparently, $RUL(0)=1$ meaning that the device has a full lifetime (100%) at the beginning $t=0$, and $RUL(N_f)=0$ meaning that the device fails when $t=N_f$.

Conventional neural network training will ignore such conditions since they are not embedded into the loss function of the neural network. In our work, we will utilize the two conditions to formulate two PINN constraints into the loss function.

## RUL estimation method using RNN

**Figure 1:**
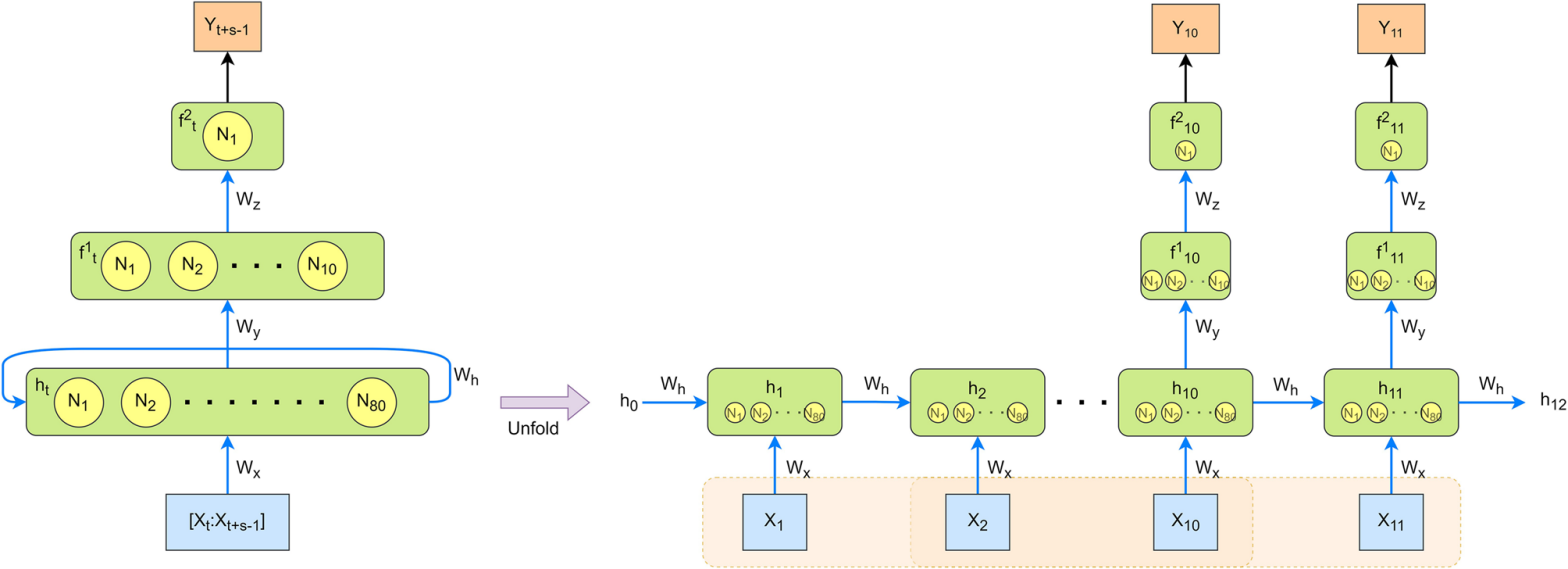
Structure of the designed RNN: The first layer, which is a recurrent layer, has 80 neurons; the second layer and the third layer are fully connected layers with 10 neurons and one neuron, respectively; and the time step *s* is set to 10.

To better handle sequence data, the neural network designed for the RUL estimation is a many-to-one type RNN, as shown in Fig. 1. There are three layers in the designed neural network. The first layer, which is a recurrent layer, has 80 neurons; the second layer and the third layer are fully connected layers with 10 neurons and one neuron, respectively. The recurrent layer can be unfolded into *s* time steps. In our model, *s* is set to 10. This means that the network takes in 10 continuous values of one input feature and produces one output.

The mathematical equations of the constructed RNN structure are given for the first layer in Eq. (2), for the second layer in Eq. (3), and for the third layer in Eq. (4). For each pair of input $[X_t:X_{t+s-1}]$ and output $Y_{t+s-1}$, $\ t,s \in {\mathbb {N}}$, we have

2 $$\begin{aligned} h_t = tanh(W_h \cdot h_{t-1} + W_x \cdot X_t + b_h), \end{aligned}$$

where $h_t$ is the first layer output, i.e., hidden state vector, at time *t*; $h_{t-1}$ is the hidden state vector at time $t-1$; $W_h$ is the weights of the hidden state vector, i.e., weights between $h_t$ and $h_{t-1}$; $W_x$ is the weights between the input and the hidden state vector; $X_t$ is the input at time *t*, and $b_h$ is the bias vector of this layer. The activation function is the hyperbolic tangent function *tanh*().

3 $$\begin{aligned} f^1_{t+s-1} = tanh(W_y \cdot h_{t+s-1} + b_y), \end{aligned}$$

where $f^1_{t+s-1}$ is the second layer output at time $t+s-1$, where $s=10$ is the time step of RNN; $W_y$ is the weights between the first layer and the second layer; $b_y$ is the bias vector added in this layer. The activation function is the hyperbolic tangent function *tanh*().

4 $$\begin{aligned} Y_{t+s-1} = f^2_{t+s-1} = W_z \cdot f_{t+s-1} + b_z, \end{aligned}$$

where $Y_{t+s-1}$ is the output of the model, i.e., the output of the third layer $f^2_{t+s-1}$; $W_z$ is the weights between the second layer and the third layer; $b_z$ is the bias vector of the third layer. The activation function is the linear function.

Since RUL estimation is a regression problem, we use Mean Squared Error (MSE) to measure the loss during network training. Let $E_{residual}=Y-{\hat{Y}}$ be the difference between the labeled value *Y* (ground truth) and predicted value ${\hat{Y}}$. The loss function of the RNN, $E_{RNN}$, can be written in the form of ordinary least squares as follows.

5 $$\begin{aligned} E_{RNN}=MSE(E_{residual})=\frac{1}{n}\sum _{i=0}^{n-1}(Y_i-\hat{Y_i})^{2}, \end{aligned}$$

where *n* is the number of training samples.

## RUL estimation method with PINN

After introducing the baseline RNN for RUL estimation and its loss function, we present our loss function with physics-informed regularization, $E_{PINN}$, which is defined as follows.

6 $$\begin{aligned} E_{PINN}{} & {} =(1-\alpha )\cdot \frac{1}{n}\sum _{i=0}^{n-1}(Y_i-\hat{Y_i})^{2}\nonumber \\{} & {} \quad +\alpha \cdot \frac{\gamma }{n-1} \sum _{i=1}^{n-1}[ReLU(\hat{Y_i}-{\hat{Y}}_{i-1})]^{2}\nonumber \\{} & {} \quad +\beta \cdot \frac{1}{n}\{\sum _{i=0}^{n-1}[ReLU(-{\hat{Y}}_i)]^{2}+\sum _{i=0}^{n-1}[ReLU({\hat{Y}}_i-1)]^{2}\}, \end{aligned}$$

where $Y_i$ denotes the ground truth of normalized RUL; $\hat{Y_i}$ denotes the predicted RUL; $\alpha$, $\beta$ and $\gamma$ are hyper-parameters to control the weights of three constraints; ReLU is the rectified linear activation function, $ReLU(x)=max(0, x)$. The loss function contains three parts: The first part is the error between the prediction and the label; the second part is the monotonic decreasing constraint, and the third part is the boundary constraint.

- Ordinary least squares (OLS). OLS is the common least squares measure for minimizing $E_{residual}=Y-{\hat{Y}}$, which is the distance between the labeled value *Y* and predicted value ${\hat{Y}}$. The loss is the mean of squared differences between them and can be defined as
  7 $$\begin{aligned} MSE(E_{residual}) = \frac{1}{n}\sum _{i=0}^{n-1}(Y_i-\hat{Y_i})^{2}. \end{aligned}$$
  This part is the same as that for RNN, Eq. (5).
- Monotonic decreasing constraint (MDC). In RUL estimation, one physical rule is that the RUL should only decrease over time. The previous predicted RUL ${\hat{Y}}_{i-1}$ should be larger than the current predicted RUL $\hat{Y_i}$, and the loss would otherwise be $\hat{Y_i}-{\hat{Y}}_{i-1}$. Mathematically this can be written as follows.
  8 $$\begin{aligned} E_{MDC} = {\left\{ \begin{array}{ll} \hat{Y_i}-{\hat{Y}}_{i-1}, &{} \hat{Y_{i}}>{\hat{Y}}_{i-1}\\ 0, &{} \text {otherwise} \end{array}\right. } \end{aligned}$$
  which can be conveniently expressed as $ReLU(\hat{Y_i}-{\hat{Y}}_{i-1})$. We use this formulation to make sure it contributes to the error only when $\hat{Y_{i}}$ is larger than ${\hat{Y}}_{i-1}$. The MDC loss can thus be defined as follows.
  9 $$\begin{aligned} MSE(E_{MDC}) = \frac{1}{n-1}\left[ \sum _{i=1}^{n-1}ReLU(\hat{Y_i}-{\hat{Y}}_{i-1})\right] ^{2} \end{aligned}$$
- Boundary condition constraint (BCC). The boundary condition for normalized RUL is $\hat{Y_{i}}\in [0,1]$. The error occurs only when the estimates go beyond the boundary conditions. For each predicted $\hat{Y_{i}}$, the error due to violating the boundary conditions can be written as follows.
  10 $$\begin{aligned} E_{BCC} = {\left\{ \begin{array}{ll} -\hat{Y_{i}}, &{}\hat{Y_{i}}<0\\ 0, &{}0\le \hat{Y_{i}}\le 1\\ \hat{Y_{i}}-1, &{}\hat{Y_{i}}>1 \end{array}\right. } \end{aligned}$$
  which can be concisely expressed as $ReLU(-{\hat{Y}}_i) + ReLU({\hat{Y}}_i-1)$. We use this formulation to capture the BCC loss, which can thus be defined as follows.
  11 $$\begin{aligned} MSE(E_{BCC}) = \frac{1}{n}\sum _{i=0}^{n-1}[ReLU(-{\hat{Y}}_i)]^{2}+\frac{1}{n}\sum _{i=0}^{n-1}[ReLU({\hat{Y}}_i-1)]^{2} \end{aligned}$$

Now we have three components constituting the customized loss function for PINN. OLS is responsible for minimizing the distance between the predicted and labeled RUL, MDC is to enhance the decreasing trend of the predicted RUL curve, and BCC punishes the predicted value exceeding the boundaries. Instead of simply combining them, we introduce weights ($\alpha$, $\gamma$, $\beta$) to the three terms to control their influence on the total loss. Briefly, we can write Eq. (6) as follows.

12 $$\begin{aligned} E_{PINN} = (1-\alpha )\cdot MSE(E_{residual}) + \alpha \cdot \gamma \cdot MSE(E_{MDC}) + \beta \cdot MSE(E_{BCC}). \end{aligned}$$

The purposes of the three parameters and their tuning principles are explained as follows.

- $\alpha$ is used to proportionally balance the error contributions between OLS (the residual error) and MDC. When we tweak $\alpha$, the contributions of OLS and MDC change with the same proportion. If we increase $\alpha$, the contribution of OLS will decrease while the contribution of MDC will increase.
- $\gamma$ is introduced to set the loss values of OLS and MDC on the same scale, such that we can jointly control the two parts in proportion.
- $\beta$ is set to control the weight of BCC to the total error. It is not tuned in proportion to OLS and MDC, because the BCC contributes to the total error only when the estimation results go across the boundary conditions (larger than 1, less than 0).
- If both $\alpha$ and $\beta$ are equal to 0, the loss function represents the case for the baseline neural network without constraints or physical rules inserted.

By tuning the three parameters, we can flexibly control the proportions of the three error terms while minimizing the total estimation error.

We would note that (1) Physics-Informed Neural Network (PINN) is not an independent NN but a technique that utilizes physical rules to strengthen the underlying NN. It is built on top of an underlying NN. PINN is a general term applicable to all kinds of underlying NNs. Depending on the underlying NN, it may be precisely termed PI-RNN (Physics-Informed RNN), if the underlying NN is RNN; it may be precisely termed PI-LSTM (Physics-Informed LSTM), if the underlying NN is LSTM. The loss function, $E_{PINN}$, is a general formulation for RUL estimation. It is not bound to a particular type of NN. In the next section, we apply this formulation to vanilla RNN and LSTM, leading to PI-RNN and PI-LSTM, respectively. (2) The spirit of PINN is to regularize the underlying NN through physical rules associated with the problem under study. This is done by adding additional terms in the NN’s loss function so that the learning algorithm can produce outputs that are more reasonable. The original PINN was developed to solve problems with Partial Differential Equation (PDE) based physical rules, but the spirit of PINN is *regularization*, i.e., formulating soft constraints into the loss function based on physical rules. The physical rules may be represented by PDEs, and might not be able to be represented by PDEs. While the original PINN is limited to the former, our work expands its scope to cover the latter. As such, our work follows the spirit of PINN and makes the original PINN more generalized.

## Results and discussion

### Experimental setup

In our experiments, we evaluate the performance of our PINN-based RUL estimation against RNN-based RUL estimation. We use the full IGBT degradation aging dataset from NASA^38^. When applying our PINN formulation, we consider two types of baseline underlying RNNs: vanilla RNN and LSTM. We will detail both in-sample and out-of-sample estimation performance for vanilla RNN, and report out-of-sample estimation performance for LSTM.

- In-sample estimation: The training data and testing data use samples from the same device or the whole set of devices. The purpose is to evaluate the model’s learning performance.
- Out-of-sample estimation: The training data and testing data use samples from different devices. The purpose is to evaluate the model’s generalization performance. In the meanwhile, we look into how the two PINN physical constraints influence the model’s learning and performance.

For network training, we employed the well-known Adaptive moment estimation (Adam) algorithm^40^, which is an improved version of stochastic gradient descent optimization algorithm. As evaluation metrics, we use both Mean Squared Error (MSE) and coefficient of determination called $R^2$ score, which are commonly used criteria to measure the performance of regression problems. $R^2$ score is a statistic that provides another measure of goodness of fit. It is the proportion of variance in the dependent variable that is explained by the model. It is defined as follows.

13 $$\begin{aligned} R^2 = 1- \frac{\sum _{i=0}^{n-1}(Y_i-\hat{Y_i})^{2}}{\sum _{i=0}^{n-1}(Y_i-{\bar{Y}})^{2}} \end{aligned}$$

where $Y_i$ denotes the ground truth of RUL; $\hat{Y_i}$ denotes the predicted RUL; ${\bar{Y}}$ is the mean value of $Y_i$; *n* is the number of samples. It can measure the proportion of the variation as a percentage which makes it easier to compare different models. The best score is 1.0 indicating the predicted values and labels are perfectly matched. The score is 0 if $\hat{Y_i}={\bar{Y}}$ meaning that the model returns a constant estimate equal to the mean value of labeled true values. If the model is worse than that, it would be negative.

Since we use $\alpha$ to balance the losses between ordinary and monotonic decreasing errors, we need to make sure that their error contributions are on the same scale. In the experiment, we added a weight of 0.1 to the monotonic decreasing constraint. This means that $\gamma =0.1$ in Eq. (6).

### The NASA dataset and pre-processing

#### The NASA IGBT dataset

The IGBT dataset is an open-source dataset from NASA Prognostics Center of Excellence (PCoE) Data Set Repository^38^. The type of device is International Rectifier IRG4BC30KD IGBT with 600V/15A current rating in TO220 package . The data were collected from an IGBT thermal overstress experiment, where a square signal was applied at the IGBT gate and parameters like gate-emitter voltage ($V_{ge}$), collector-emitter voltage ($V_{ce}$), and collector-emitter current ($I_{ce}$) were recorded^39^.

The failure mode is transistor latch-up (not package failure). The latch-up failure leads to a high current between the collector and the emitter, which can be captured by the drastic drop in the collector-emitter voltage ($V_{ce}$). The latch-up failure itself will not cause immediate damage to the IGBT; it is the latch-up caused thermal runaway that will damage the device. However, in the experiment^39^, a temperature threshold controller was used to prevent this damage from happening by turning off the load power supply to terminate the test once the thermal runaway (temperature exceeding threshold) occurred. In this way, the device can still be functional after the latch-up failure point but the failure mechanism was simulated.

As with previous studies^2,5,33^, we consider the collector-emitter voltage ($V_{ce}$) as the precursor signal. We regarded the abrupt drop in collector-emitter voltage ($V_{ce}$) as the device failure point. Only four devices were given in the dataset, starting from device 2 to device 5. Please note that we keep the same device numbering in the paper as the original dataset.

#### Data preprocessing

**Figure 2:**
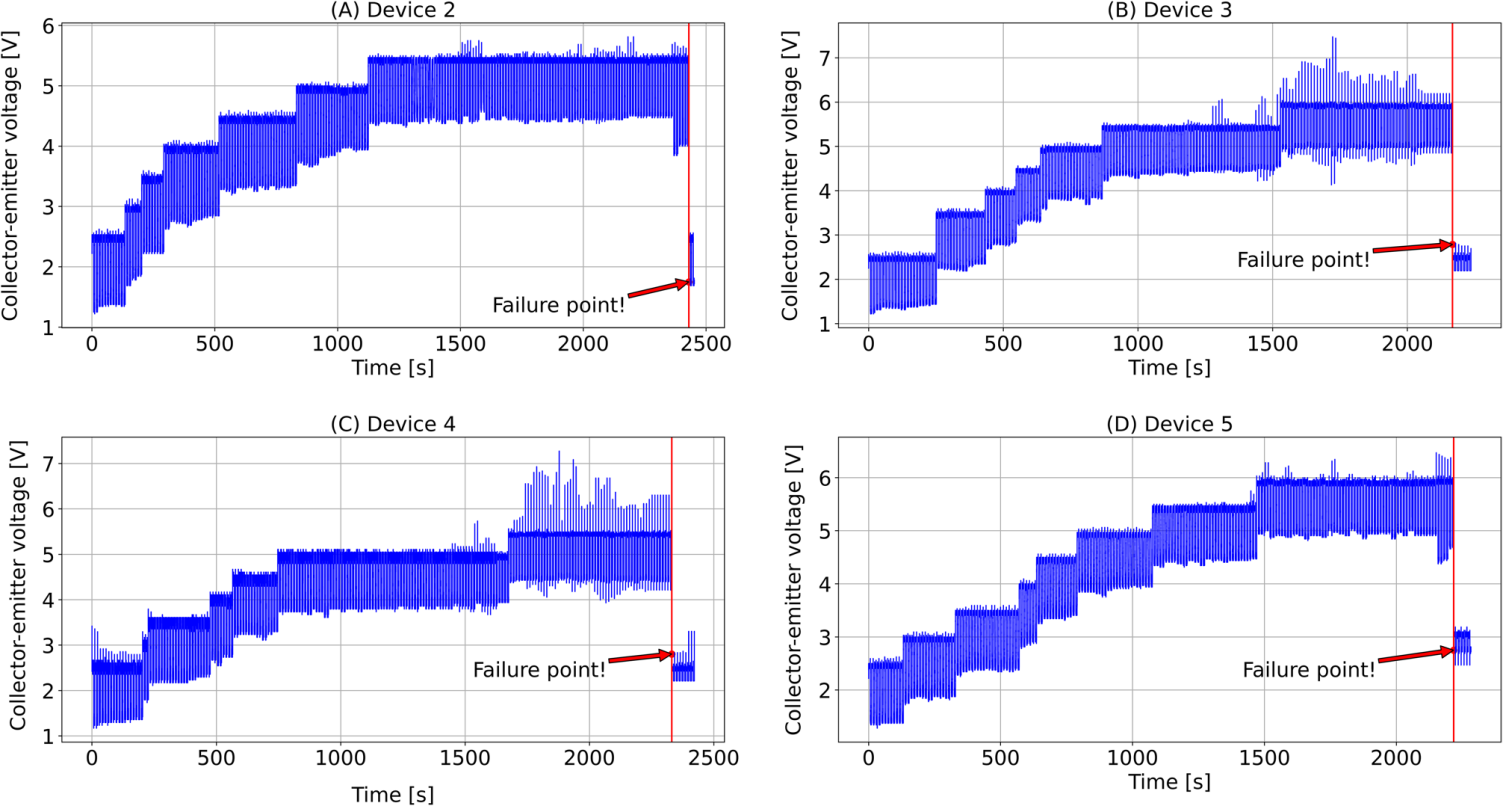
The original collector-emitter voltage ($V_{ce}$) data of (**A**) device 2, (**B**) device 3, (**C**) device 4, (**D**) device 5 with failure points labeled.

Referring to the data acquisition experiment^39^, we identified the failure points of four devices from the precursor signal ($V_{ce}$) and cut off data after the devices failed. The original $V_{ce}$ signals of all four devices are visualized in Fig. 2. We used the following three steps to preprocess the data set.

**Figure 3:**
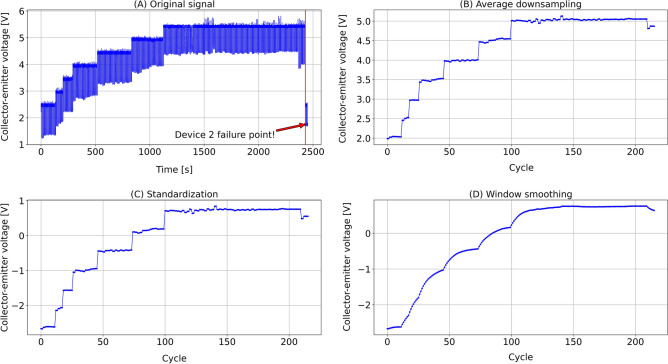
Data preprocessing of collector-emitter voltage ($V_{ce}$) of device 2: (**A**) Original signal, (**B**) Average downsampling, (**C**) Standardization, (**D**) Window smoothing.

**Figure 4:**
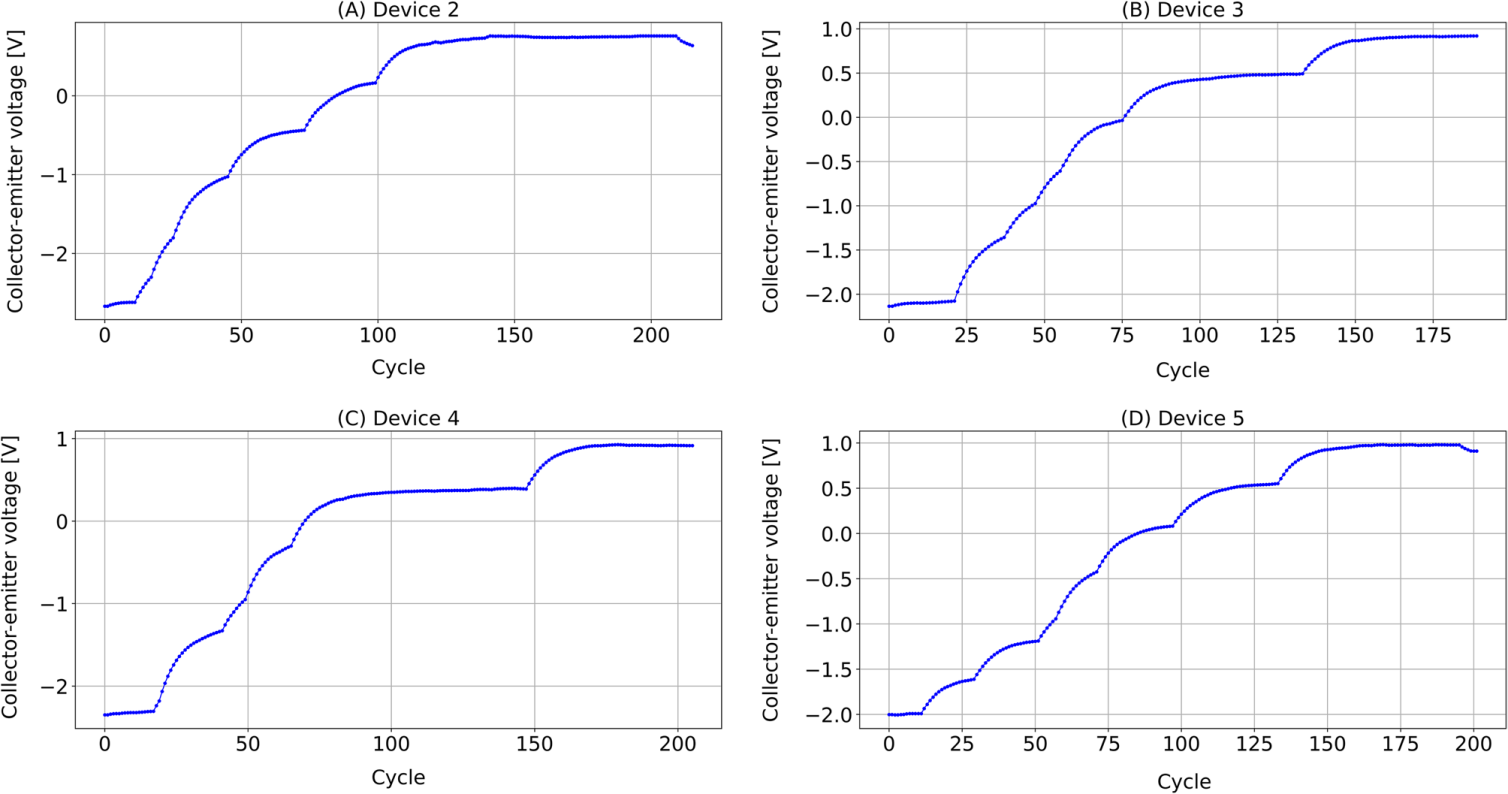
The preprocessed collector-emitter voltage ($V_{ce}$) data of (**A**) device 2, (**B**) device 3, (**C**) device 4, (**D**) device 5.

*Average downsampling* Since the square signal was applied at the gate of the IGBT device in the experiment, the collector-emitter voltage was in the form of square wave as well. Therefore, we downsampled the raw data to one sample in one square wave cycle by calculating the average value of this cycle.

*Standardization* A zero mean ($\mu =0$) and unit standard deviation ($\sigma =1$) were used to standardize the downsampled dataset so that the prediction did not depend on the exact data values.

*Window smoothing* Exponential Moving Average (EMA) algorithm^41^ was applied to smooth the standardized data, facilitating the neural network to learn and fit. In contrast to Simple Moving Average (SMA) algorithm, EMA puts more weight on the most recent data points. The EMA function is given as follows:

14 $$\begin{aligned} y_t = \frac{x_t + (1-\theta ) \cdot x_{t-1} + (1-\theta )^2 \cdot x_{t-2} +\cdots + (1-\theta )^t \cdot x_0}{1 + (1-\theta ) + (1-\theta )^2 +\cdots + (1-\theta )^t}, \end{aligned}$$

where $x_t$ denotes the original series; $y_t$ denotes the smoothed series; $\theta$ denotes the decay factor given by $\theta = 2/(span + 1)$, where *span* is set to 15 as the width of the sliding window applied to the original series.

As an example, Fig. 3A–D shows the original data, after the average down-sampling, after the standardization, and after the window smoothing, respectively. The smoothed dataset for all four devices is drawn in Fig. 4.

### In-sample estimation performance: RNN versus physics-informed RNN (PI-RNN)

**Figure 5:**
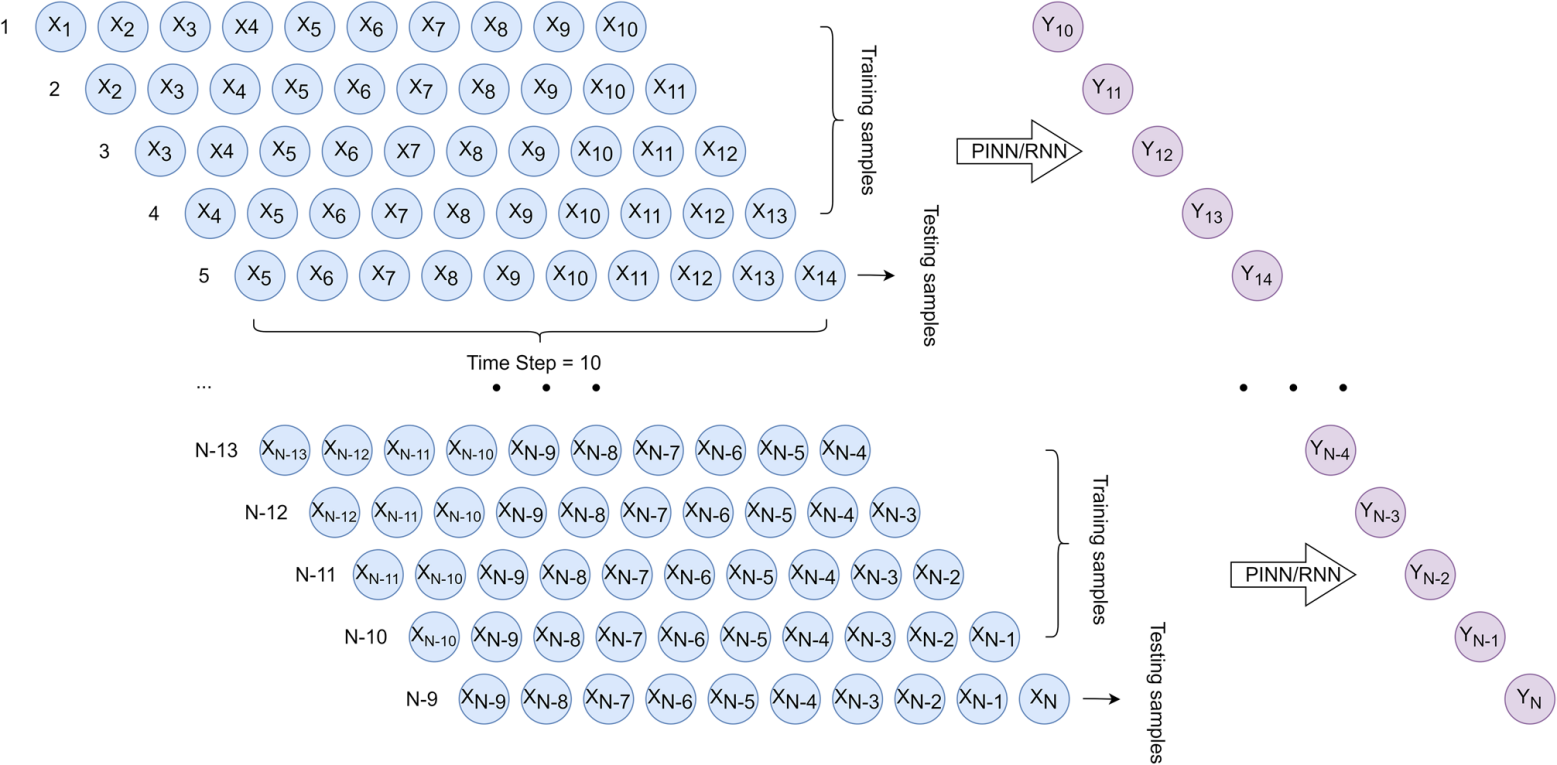
Data splitting (80% training and 20% testing) for in-sample performance evaluation.

In the in-sample experiment, we trained RNN models for each individual device and four devices as a whole, with 80% of data for training and 20% of data for testing. Since our monotonic decreasing condition needs the information about previously predicted RUL to calculate the loss, we should keep the sequence order of data samples. To this end, for every 5 samples, the last one was extracted and they were concatenated as test data as illustrated in Fig. 5.

**Table 1:** In-sample RUL estimation performance for individual/all devices ($\alpha =0.1$, $\beta =1$).

Train/Inference device(s)	MSE $\times 10^{4}$		MSE $\times 10^{4}$		Improvement		$R^2$ score		$R^2$ score	
	RNN		PI-RNN		MSE ($\%$)		RNN		PI-RNN	
80% train : 20% test	Train error	Test error	Train error	Test error	Train	Test	Train	Test	Train	Test
2	28.3204	30.7122	22.4979	26.7838	20.56	12.79	0.9629	0.9597	0.9705	0.9649
3	5.5014	7.0736	1.4082	1.5279	74.40	78.40	0.9927	0.9906	0.9981	0.9980
4	5.4812	6.0971	2.7370	2.9855	50.07	51.03	0.9928	0.9919	0.9964	0.9960
5	5.5408	6.1395	2.7198	2.8099	50.91	54.23	0.9927	0.9918	0.9964	0.9963
2, 3, 4, 5	31.7129	32.9313	17.4423	19.2403	45.00	41.57	0.9583	0.9564	0.9771	0.9745
Average	15.311	16.591	9.3610	10.6695	38.86	35.69	0.980	0.978	0.988	0.986

**Figure 6:**
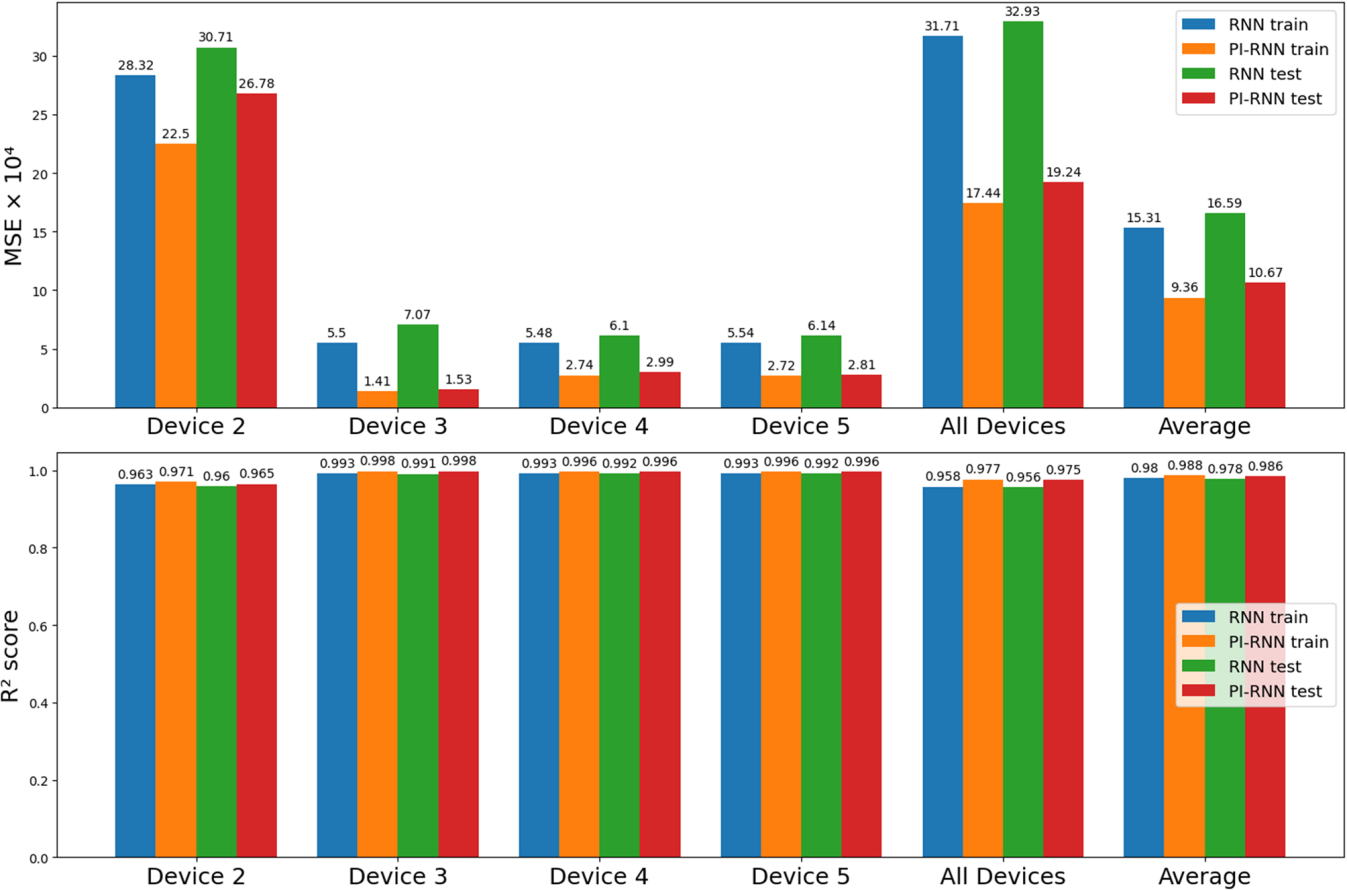
In-sample RUL estimation performance comparison of RNN and PI-RNN ($\alpha =0.1$, $\beta =1$).

Table 1 and Fig. 6 compare the in-sample performance of RNN and PI-RNN with $\alpha =0.1$ and $\beta =1$. We can see that PI-RNN achieves a better MSE performance than RNN in both training and testing with 38.86% and 35.69% improvements, respectively. PI-RNN has the most significant MSE improvement on Device 3, with 74.4% for training and 78.4% for testing. The minimum MSE performance improvement appears on Device 2, with 20.56% for training and 12.79% for testing. Compared with other devices, Device 2 has a much larger error when training and testing with PI-RNN and RNN. This is due to the vague $V_{ce}$ feature of Device 2 in the second half of its RUL (see Fig. 4). With all devices as a whole, PI-RNN reduces the training error by 45% and the testing error by 41.57%. For $R^2$ score, both PI-RNN and RNN achieve comparable performance, and PI-RNN has a slightly better $R^2$ score than RNN.

### Out-of-sample estimation performance: RNN versus physics-informed RNN (PI-RNN)

To evaluate the out-of-sample performance, we employed 4-fold cross validation whereas 4 cases were set up as listed in Table 2, the left three columns. For each case, 3 of 4 devices were selected for training and 1 for testing. We first evaluate the impact of the monotonic decreasing condition, then the impact of the boundary condition, and finally the impact of both conditions.

**Table 2:** Out-of-sample RUL estimation performance with 4-fold cross-validation.

Group			MSE$\times 10^{3}$		MSE$\times 10^{3}$		Improvement		$R^2$ score		$R^2$ score	
			RNN		PI-RNN		MSE(%)		RNN		PI-RNN	
Case	Train set	Test set	Train error	Test error	Train error	Test error	Train error	Test error	Train error	Test error	Train error	Test error
1	2, 3, 4	5	7.068	14.990	4.258	2.467	39.8	83.5	0.907	0.802	0.944	0.967
2	2, 3, 5	4	7.201	1.658	5.880	1.476	18.3	11.0	0.905	0.978	0.923	0.980
3	2, 4, 5	3	2.923	6.631	2.752	5.768	5.8	13.0	0.960	0.913	0.964	0.924
4	3, 4, 5	2	5.922	8.732	4.513	5.876	23.8	32.7	0.922	0.885	0.940	0.923
Average			5.784	8.014	4.352	3.903	24.7	51.3	0.924	0.895	0.943	0.949

**Figure 7:**
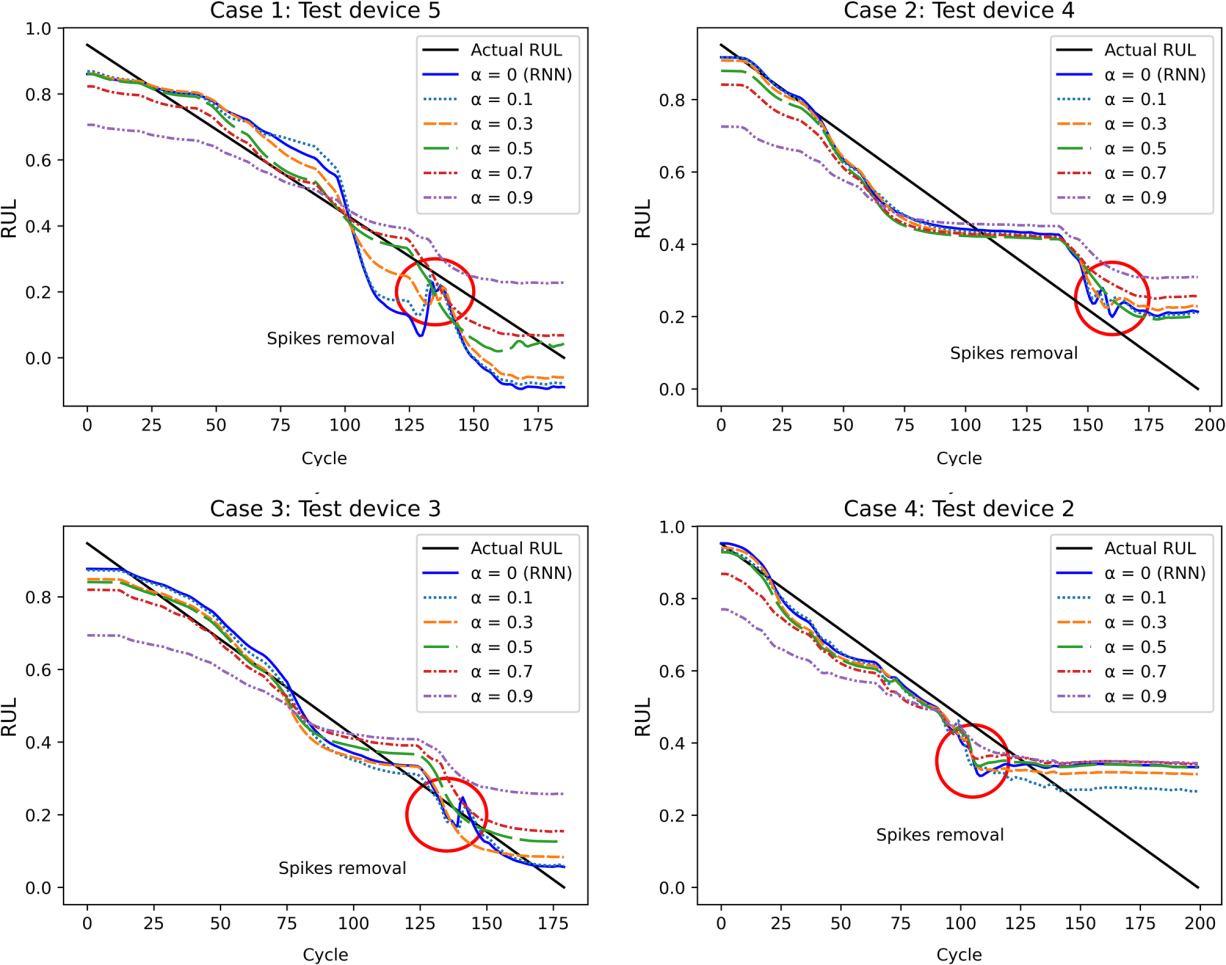
Influence of monotonic decreasing condition ($\alpha = 0, 0.1, 0.3, 0.5, 0.7, 0.9$, $\beta =0$).

**Figure 8:**
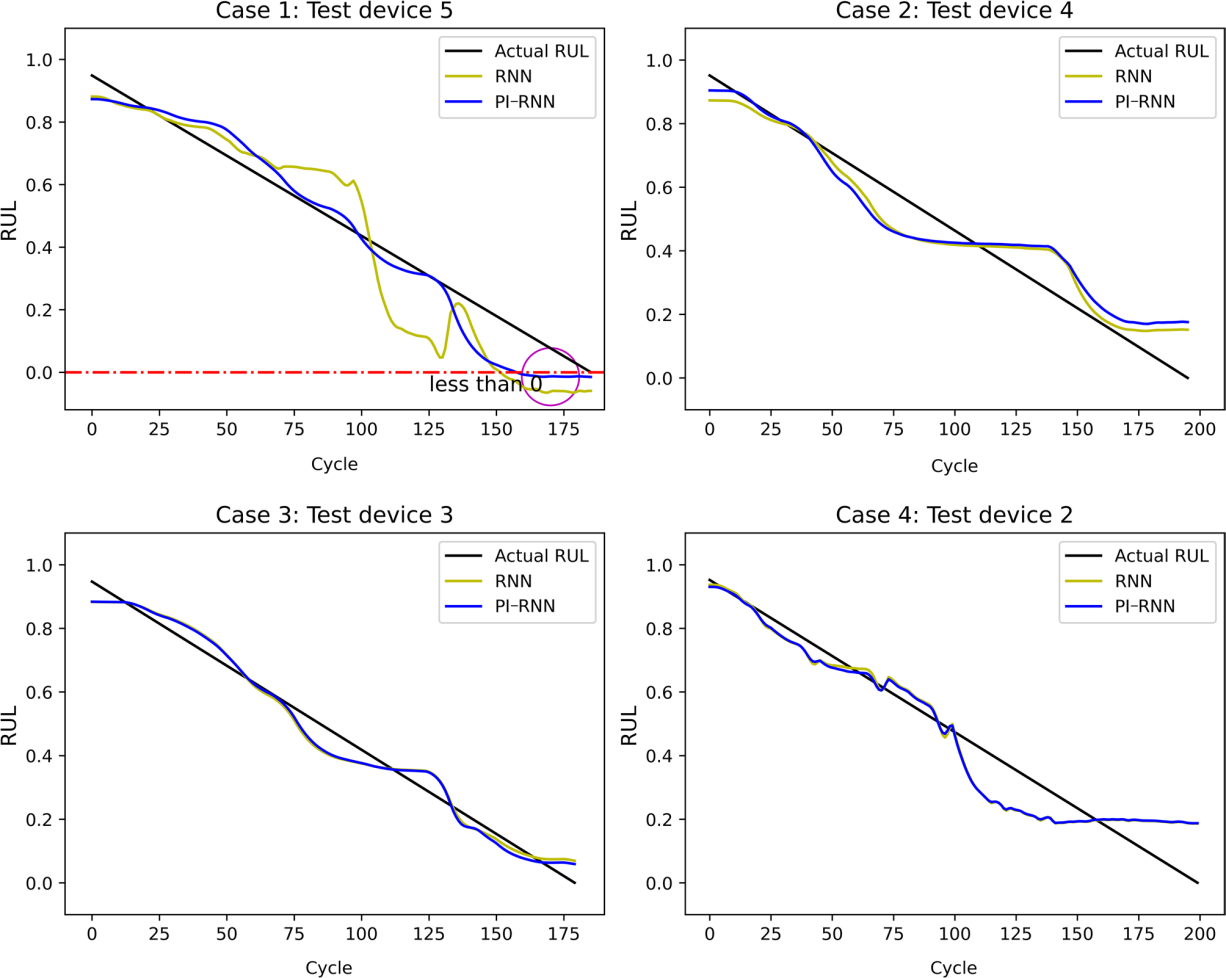
Influence of boundary condition on RUL estimation by RNN and PI-RNN ($\alpha$ = 0, $\beta$ = 100).

#### Influence of monotonic decreasing condition

Four groups of experiments were conducted by tweaking the parameter $\alpha$ for weighing the monotonic decreasing condition to tune its contribution to the total loss function. We trained the PI-RNN with $\alpha = 0, 0.1, 0.3, 0.5, 0.7, 0.9$ and the results of four cases are shown in Fig. 7. Note that, when $\alpha = 0$, it means the absence of the monotonic decreasing constraint, thus the model is RNN.

For all four cases, the slope of the predicted RUL becomes flatter as $\alpha$ increases. Without the monotonic decreasing constraint ($\alpha =0$), the predicted RUL by RNN could go up which is impossible in real life. However, when increasing the weight of the monotonic decreasing constraint, i.e., increasing the value of $\alpha$, we could eliminate the spikes on the curves, which are marked with the red circles in Fig. 7.

#### Influence of boundary condition

The boundary condition intends to limit the predictions within the value range from 0 to 1. PI-RNN with only boundary condition but without monotonic decreasing condition ($\alpha =0$, $\beta =100$) is compared with the original RNN in Fig. 8. In Cases 2, 3, and 4, as the RUL predicted by the original RNN always ranges from 0 to 1, the boundary condition cannot make much difference. This is expected because the underlying boundary condition is already fulfilled. However, In Case 1, the original RNN predicts some RUL values less than 0 near the end of its lifetime, which is contrary to the boundary condition. After applying the boundary condition constraint, the predictions converge to 0. Also, the loss on the testing is reduced by 76% from $14.99\times 10^{-3}$ to $3.59\times 10^{-3}$.

#### Influence of both physical conditions

**Figure 9:**
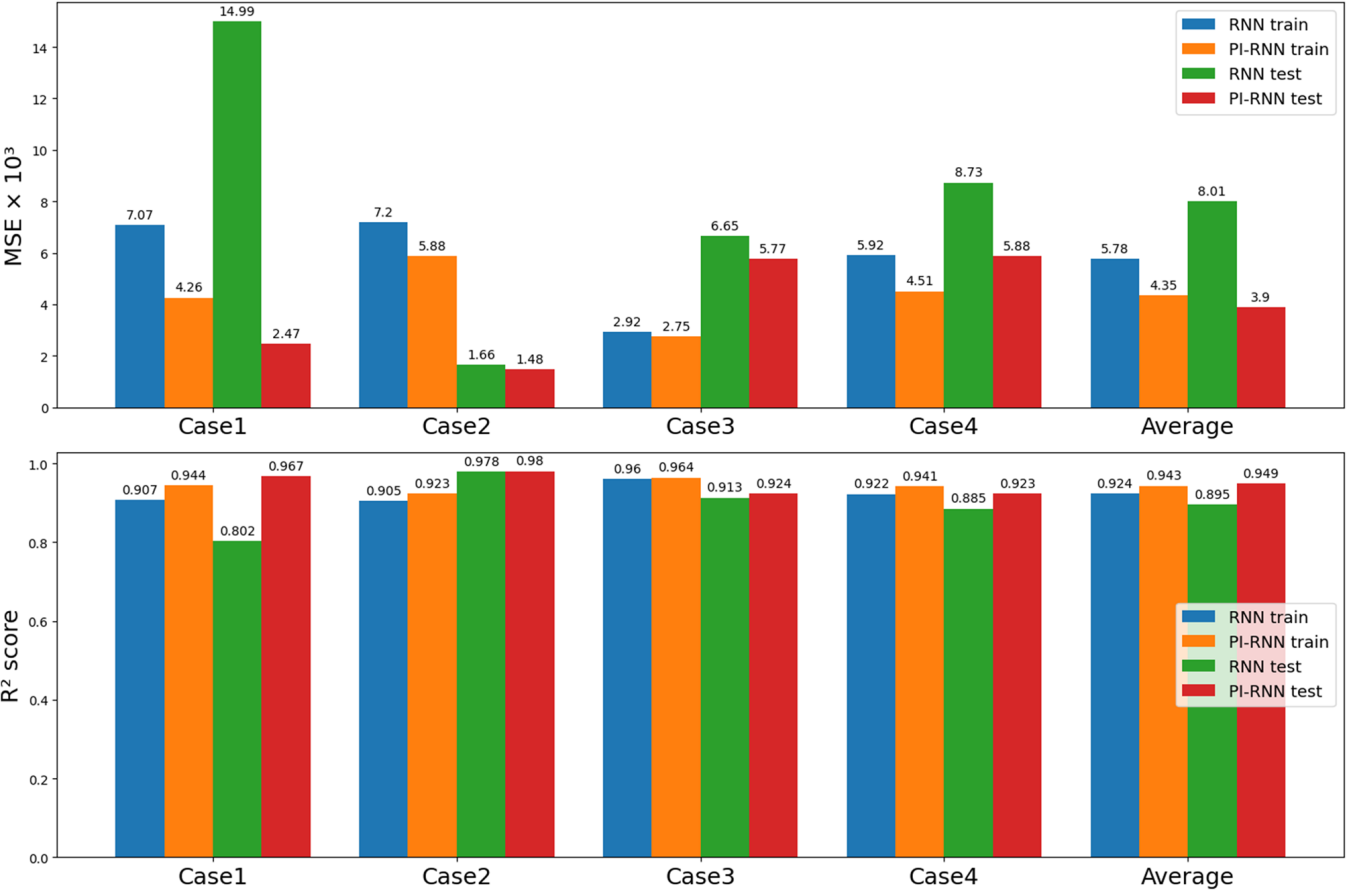
Out-of-sample RUL estimation performance comparison of RNN and PI-RNN ($\alpha =0.1$, $\beta =100$).

When applying both physical conditions, PI-RNN can improve the performance, in both MSE and $R^2$ score, of training and testing in all 4 cases. As shown in Table 2 and Fig. 9, by applying PI-RNN with the parameters, $\alpha = 0.1$ and $\beta = 100$, MSE is improved by 24.7% from $5.784 \times 10^{-3}$ to $4.352 \times 10^{-3}$ in training and 51.3% in testing from $8.014 \times 10^{-3}$ to $3.903 \times 10^{-3}$ for all 4 cases on average. The maximum improvement occurs in Case 1, for which PI-RNN improves the training MSE by 39.8% from $7.068\times 10^{-3}$ to $4.258\times 10^{-3}$ and the testing MSE by 83.5% from $14.99\times 10^{-3}$ to $2.467\times 10^{-3}$. The minimum improvement occurs in Case 3, for which PI-RNN improves the training MSE by 5.8% and the testing MSE by 13%. Furthermore, PI-RNN increases $R^2$ score on average by 2% from 0.924 to 0.943 in training, and by 6% from 0.895 to 0.949 in testing. In conclusion, PI-RNN is able to improve the generalization capability of the baseline RNN as a result of regularizing the RNN to conform to the two physical conditions.

We would note that, in our experiments, the RUL tends to be constant at the end. This is because the precursor signal reaches a final constant level before failure. To decide whether the device is reaching its end of lifetime, we may consider a refined strategy to (1) differentiate and determine multiple device health stages, e.g., healthy, sub-healthy, pre-failure, and failure; (2) When the pre-failure health stage is reached, a polynomial model may be fitted instead to estimate RUL for the rest of lifetime. Since this work focuses on PINN for RUL estimation, this can be a direction for future investigation.

### Out-of-sample estimation performance: LSTM versus physics-informed LSTM (PI-LSTM)

As a more powerful variant of RNN, LSTM has also been proposed for RUL estimation^3,7^. To show that our PINN formulation can also work well with LSTM, we evaluated the out-of-sample estimation performance of pure LSTM and physics-informed LSTM (PI-LSTM) with the same 4-fold cross-validation described in the previous subsection. The structure of LSTM is similar to that of RNN in the previous experiments: 1 neuron in the input layer, 80 LSTM cells in the first hidden layer, 10 neurons in the second hidden layer, and 1 neuron in the output layer. Due to the additional three gating mechanisms (forget gate, input gate, and output gate) in an LSTM cell, it has four times the number of weight/bias parameters of a corresponding vanilla RNN unit. As a result, the total number of parameters in the LSTM is approximately four times as many as that of the RNN (27,061 parameters in the LSTM and 7,381 parameters in the RNN).

**Figure 10:**
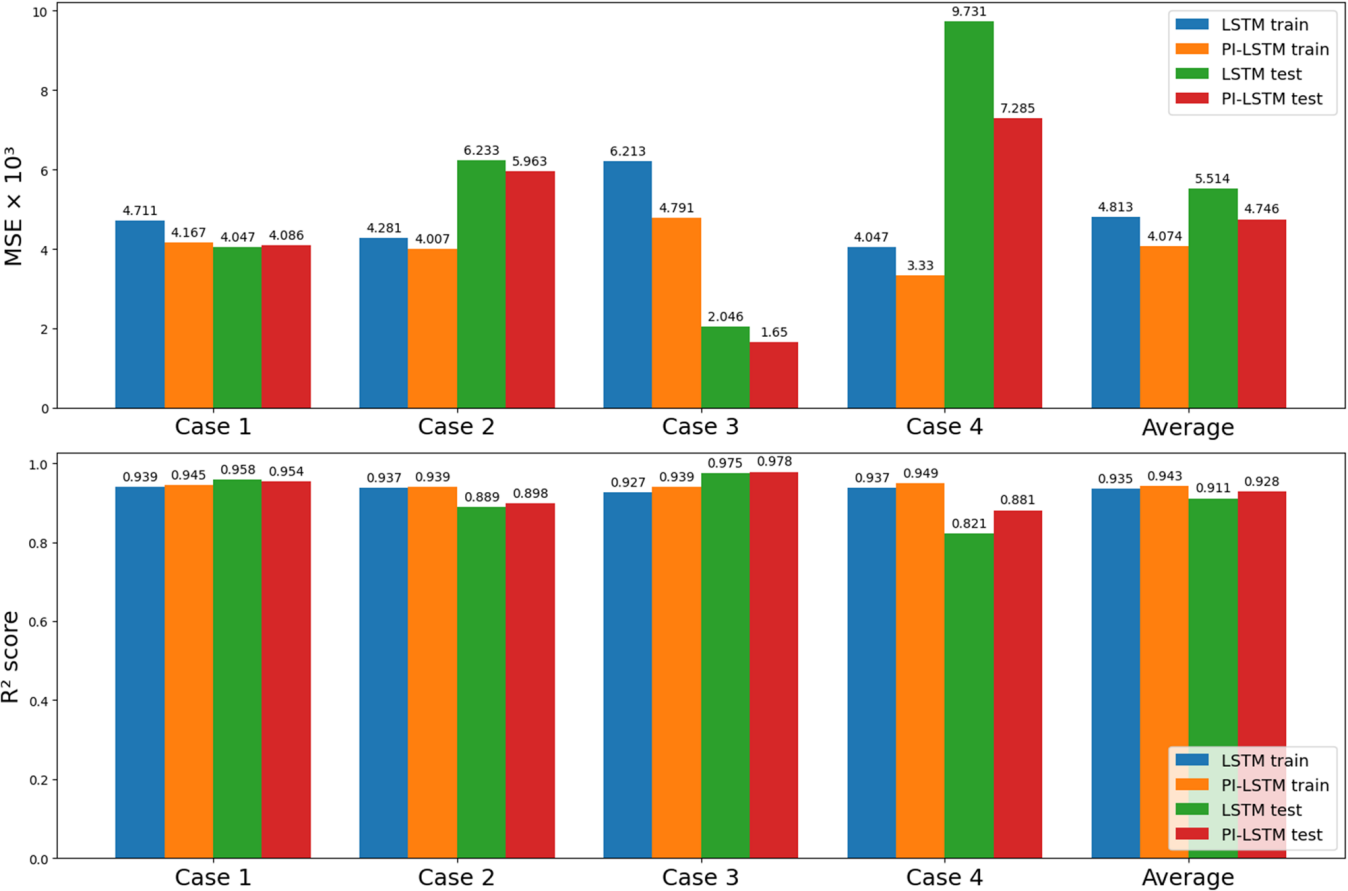
Out-of-sample RUL estimation performance comparison of LSTM and PI-LSTM ($\alpha = 0.1$, $\beta = 100$).

Both physical conditions were applied in the physics-informed LSTM with the parameters $\alpha = 0.1$ and $\beta = 100$. The MSE and $R^{2}$ score are shown in Fig. 10.

After introducing two physical conditions, the performance of the LSTM is increased in both MSE and $R^{2}$ score with only one exception, a mere 1% MSE rise of testing in Case 1. For all four cases, the MSE is improved on average by 15.3% from $4.813 \times 10^{-3}$ to $4.074 \times 10^{-3}$ in training and 13.9% from $5.514 \times 10^{-3}$ to $4.746 \times 10^{-3}$ in testing. The $R^{2}$ score is improved on average by 0.9% from 0.935 to 0.943 in training and 1.9% from 0.911 to 0.930 in testing. As a result, we can conclude that the two physical-rule based constraints work on LSTM as well.

## Conclusion

We have proposed an RUL estimation method for IGBTs using PINN. The physical rules are identified from the target RUL function and formulated as two regularization terms (monotonic decreasing and boundary conditions) in the loss function of the underlying NN. By adjusting their weighted importance, the regularization makes the trained neural network conform to a monotonic decreasing trend and removes negative values. We have applied our method to RNNs for RUL estimation using the NASA IGBT data set. In the in-sample estimation experiments with vanilla RNN, our physics-informed RNN can improve the MSE of the baseline underlying RNN on average by 38.86% in training and by 35.69% in testing. In the out-of-sample estimation experiments with vanilla RNN, our physics-informed RNN can improve the MSE of the baseline underlying RNN on average by 24.7% in training and by 51.3% in testing. In the out-of-sample estimation experiments with LSTM, our physics-informed LSTM can improve the MSE of the baseline underlying LSTM on average by 15.3% training and 13.9% in testing. This implies a large expansion of the NN models’ generalization capability. In both in-sample and out-of-sample estimation, PINN does not compromise $R^2$ score. Actually, it slightly enhances $R^2$ score in all cases. Our approach opens a new path for RUL estimation by combining data-driven with physics information, and perhaps more significantly, it can be inspiring for expanding PINN to address other non-mathematical real-life problems that need to identify and formulate physical rules into the underlying NN’s loss function for regularization.

## Data Availability

The dataset used and analyzed during the current study is available in the NASA Prognostics Center of Excellence (PCoE) Data Set Repository, Data Set 8 on Insulated-Gate Bipolar Transistor (IGBT) Accelerated Aging. https://www.nasa.gov/content/prognostics-center-of-excellence-data-set-repository.
